# Structural conditions of interprofessional collaboration in neurological early rehabilitation wards: a qualitative study

**DOI:** 10.3389/frhs.2026.1862640

**Published:** 2026-08-04

**Authors:** L. Redzewsky Faure, U. Thielhorn, A. Frangos, C.-W. Wallesch, M. Körner

**Affiliations:** 1 Medical Psychology and Medical Sociology, Faculty of Medicine, University of Freiburg, Freiburg, Germany; 2 Catholic University of Applied Sciences Freiburg, Freiburg, Germany; 3 Institute of General Practice, Medical Faculty and Medical Center, University of Freiburg, Freiburg, Germany; 4 BDH-Bundesverband Rehabilitation, Bonn, Germany; 5 Departement of Health Professions, Institute for Collaborative Practice and Leadership in Healthcare, Bern University of Applied Sciences, Bern, Switzerland

**Keywords:** interprofessional collaboration, neurological early rehabilitation, organisational culture, qualitative research, structural conditions, ward organisation, work organisation

## Abstract

**Background:**

Early neurological and neurosurgical rehabilitation (ENR, phase B) forms the transition from acute treatment to further rehabilitation and requires close integration of acute medical and rehabilitative measures. Interprofessional collaboration (IPC) is a central component of rehabilitative care according to the ICF (International Classification of Functioning, Disability and Health) model. Previous research has focused on individual and team competencies to promote IPC. Recent studies suggest that IPC is also influenced by structural conditions. Against this background, the study examines which structural elements of ENR wards enable interprofessional work organisation.

**Materials and methods:**

The study employed a qualitative design, including group discussions with interprofessional teams across five neurological rehabilitation facilities with ENR wards in Germany. Using purposive sampling, 76 employees from six professional groups and three hierarchical levels participated in 15 interprofessional group discussions, which were transcribed verbatim. The evaluation was carried out as a qualitative structured content analysis with deductive and inductive categories according to Kuckartz and Rädiker based on Sackmann's cultural model.

**Results:**

The results show three core structural dimensions that shaped interprofessional work organisation in ENR: (1) form of therapy planning, (2) staff allocation on the ward, and (3) information exchange. From these, three types of interprofessional work organisation (agile, adaptive, prospective) were reconstructed. Particularly beneficial for the care of ENR patients in phase B is decentralised therapy planning, adapted to clinical volatility, with fixed interprofessional ward teams, and daily structured handover meetings in addition to weekly team meetings.

**Conclusion:**

The findings suggest that ward organisation should be based on the severity of the patients' condition: the greater the clinical instability, the more flexible and closely knit the interprofessional work organisation should be.

## 1 Background

Neurological-neurosurgical early rehabilitation (ENR) is a highly complex area of care that treats patients with severe damage to the central nervous system. Thanks to advances in acute and intensive care medicine, more people survive serious neurological events today, but they often remain with significant functional limitations and an unclear prognosis for rehabilitation (1, 2). Rehabilitative care in Germany is organised according to a phase model that ranges from acute treatment (phase A) to long-term care (phase F) (1). ENR (phase B) is the central transition from acute medical stabilisation to further rehabilitation (phases C, D). ENR patients require monitoring, often have variable clinical courses, and need both acute medical and rehabilitative measures. These are based on the International Classification of Functioning, Disability and Health (ICF) (2–4).

Care in the ENR requires continuous and highly coordinated interprofessional collaboration (IPC). IPC is understood to mean cooperative collaboration under a common goal involving at least two professional groups (5). This is structurally supported by statutory requirements such as interprofessional early rehabilitation teams and weekly interprofessional team meetings (1, 6).The literature shows that IPC is considered a key success factor in rehabilitation (5, 7). At the same time, Wade (8) and Franz et al. (6) indicate that formal exchange formats, especially under time pressure, are not sufficient to consistently implement interprofessional decisions.

Research increasingly emphasises that IPC does not only arise at the level of individual competencies or internal team processes. Rather, organisational framework conditions are decisive in determining whether IPC can be effectively implemented (9–11). Supportive structural elements include shared documentation and communication systems, regular interprofessional exchange formats, physical proximity of professional groups, and sufficient human and time resources (12–15). Structural deficits, on the other hand, can significantly impair IPC (16, 17).

To date, empirical evidence on the structural conditions shaping interprofessional work organisation in ENR remains limited. Franz et al. (6) provided important initial insights into interprofessional collaboration in neurological rehabilitation; however, the study focused primarily on nursing and therapy professionals and addressed organisational structures only to a limited extent. Consequently, there is still a lack of cross-professional research examining how structural conditions specifically shape and enable interprofessional work organisation in phase B ENR.

This research gap is significant because structures in organisations are changeable and can be shaped by strategic measures (18). Management plays a central role in this, particularly developing processes, spatial and personnel conditions, and formal coordination mechanisms (19, 20). Improved IPC, therefore, requires both structural and cultural adjustments in the organisational context.

The present study addresses this gap and examines how ward structures shape interprofessional work organisation in ENR. It is based on a partial evaluation of a more comprehensive study on organisational culture in ENR and focuses exclusively on structural determinants. The following research question is explored: “Which structural elements of neurological early rehabilitation wards enable effective interprofessional work organisation?” The aim of the study is to identify and describe structural determinants that shape interprofessional work organisation in ENR.

## 2 Materials and methods

### 2.1 Study design

This study used a qualitative, exploratory design and conducted group discussions with interprofessional teams who had work experience in ENR wards. The theoretical framework was based on Sackmann's (21, 22) concept of organisational cultural knowledge dimensions. Organisational structure was understood as an integral part of organisational culture. By collecting organisational cultural knowledge, conclusions could be drawn about the structural conditions of the wards and the IPC work organisation. The following results represent partial findings from the first author's dissertation (23).

### 2.2 Study setting

The study population comprised five neurological rehabilitation facilities in Germany. The facilities were distributed throughout the country; four were in small towns in rural areas, and one was in a district town. All locations had ENR wards including intensive care and artificial ventilation that care for ENR patients in phase B, with interprofessional team structures, and offer further rehabilitation services in phases C and D. Two facilities also provided acute neurological care as part of phase A.

### 2.3 Study participants

The study sample was selected purposively according to predefined inclusion criteria. Eligible participants were employees who worked, or had previously worked, on NFR wards treating phase B patients, or who had organisational or managerial responsibility for these wards. Inclusion criteria were guided by code 8-552 of the German Operation and Procedure Classification System, which defines medicine, nursing, therapy and (neuro-)psychology as core professional groups of neurological early rehabilitation teams (24). Staff from social services and discharge management were additionally included because of their close involvement in ward organisation, and hospital management (managing director, medical director, nursing director) were included due to their influence on organisational structures (Table 1).

**Table 1:** Inclusion and exclusion criteria.

Participation in the study	Criteria
Inclusion	Employees of clinics offering early neurological rehabilitation, minimum age 18, current employment, occupational groups: medicine, nursing, therapy, (neuro)psychology, discharge management/social services, business administration (management, commercial management).
Exclusion	Employees not involved in early neurological rehabilitation, Interns, All other professional groups in the clinics (e.g., clinic services, kitchen, secretarial staff, cleaning staff, laundry staff).

Three group discussions were conducted in each participating facility, resulting in 15 group discussions overall. A minimum sample of 75 and maximum of 105 participants was planned. Where possible, at least one representative from each relevant professional group was included. A larger sample was planned at staff level to capture the diversity of therapeutic professions, including physiotherapy, occupational therapy and speech therapy. (Neuro-)Psychology was considered separately because this profession was usually organised across wards (Table 2).

**Table 2:** Sampling plan for group discussions per facility.

Level	MD	M	N	T	NP	SW	Total
Hospital management	1–2	1	1	1	0	0	5
Ward management	0	1	1	1	1	1	5
Staff	0	1–2	1–2	1–3	1–2	1–2	5–11

MD, managing director; M, medical; N, nurse; T, therapist; NP, (neuro-)psychologist; SW, social worker.

The participants were recruited through hospital management, medical directors, nursing and therapy managers, ward managers, internal newsletters and hospital websites. In some cases, potential participants were approached personally by the researcher or a local key person. Participation was voluntary. Before participation, all individuals received written information on the purpose of the study, voluntary participation, the responsible institution, audio recording procedures and data protection rights. Written informed consent was obtained from all participants.

### 2.4 Data collection

Data collection took place between July 2022 and April 2023 and was conducted on site at the participating facilities. Due to the COVID-19 pandemic, data collection required additional organisational coordination. Staff shortages limited the availability of employees, and site-specific hygiene regulations had to be observed, including maximum room occupancy, physical distancing and mask requirements. The group discussions were scheduled individually with each facility, conducted by the first author, organised separately by hierarchical level, and took place once during regular working hours in meeting rooms at the respective facilities. The group discussions followed a semi-structured interview guide informed by Bohnsack's methodological approach to group discussions and first applied in a pre-test (25). Based on the pre-test, the opening stimulus was refined to the following: “I am interested in how you collaborate interprofessionally in your everyday professional practice when caring for patients. Please tell me about your shared day-to-day work at the hospital. In which areas do you work together with other professional groups? What experiences have you had with interprofessional collaboration?” The originally planned discussion duration was also reduced from 2 h to approximately 1 h, reflecting limited opportunities to release staff during routine clinical work. The pre-test material was included in the final analysis. The structure of the group discussions followed a process-oriented procedure that was reproducible despite thematic variation between discussions, thereby supporting comparability across the data material (25). During and after each discussion, the researcher prepared observation notes. The database comprised a demographic questionnaire and transcribed audio recordings of the group discussions. The transcripts and findings were not returned to the group discussion participants for validation. The data provided sufficient thematic saturation to address the research questions; therefore, no further data collection was undertaken.

### 2.5 Data analysis

The audio recordings were transcribed using a clean verbatim approach in accordance with the content-semantic transcription rules developed of Dresing and Pehl (26) immediately after each data collection. Data analysis began in 2023 after completion of the full data collection. The evaluation followed a structured qualitative content analysis, according to Kuckartz and Rädiker (27). Deductive categories were based on the knowledge dimensions of the cultural model, according to Sackmann (21, 22) and were supplemented with subcategories developed inductively from the material. MaxQDA (version 24.4.1, VERBI GmbH) and Microsoft Office were used for the analysis.

The present paper reports selected findings from the broader study of Redzewsky Faure (23). Of the 1798 coded segments in the full dataset, 1187 deductive and inductive codes were included in this paper. These codes were primarily drawn from the category's lexical knowledge—organisational structure and action knowledge—task accomplishment and interpersonal relationships (Table 3). The coding framework comprised four hierarchical levels: the first two levels were deductively derived from Sackmann's (22) model, while the third and fourth levels were inductively differentiated from the empirical data. The complete coding framework of the original study is provided in Supplementary Material.

**Table 3:** Deductive categories according to Sackmann (21, 22) and inductive categories differentiated from the data.

Level	Theme	Explanation
1.	Dictionary knowledge	Commonly known knowledge about definitions and descriptions. It refers to the “what is” in a particular cultural environment.
1.2.	Organisational structure	Referring to structural aspects and formal procedures of the organisation. These create the conditions for achieving the main objective and implementing the overall strategy.
1.2.1.	Hospital network level	Refers to statements concerning the hospital's non-profit ownership and the significance of its membership in the hospital network.
1.2.2.	Hospital level	Refers to the structure of collaborative formats within the hospital that extend across wards and professional groups.
1.2.3.	Ward level	Refers to the structural organisation of wards within the institution.
1.2.3.1.	Ward configuration	Refers to how the ward is staffed and how patients are allocated according to the severity of their condition.
1.2.3.2.	Therapy planning	Refers to the formal organisation of therapy on the ward, including how therapeutic interventions are planned,
1.2.3.3.	Ward meetings	Refers to formally scheduled interprofessional meeting formats on the ward, as well as to the absence of such formats.
1.2.3.4.	Team meetings	Refers to the OPS 8-552 structural requirement for a weekly interprofessional team meeting based on the ICF framework. During these meetings, weekly patient goals are defined and further care is discussed. The category includes statements concerning the organisation, implementation, challenges, and perceived benefits of these team meetings.
1.2.3.5.	Within professional groups	Refers to meeting formats, descriptions, and specific characteristics within individual professional groups.
1.2.3.6.	Ward-independent professional groups	Refers to structural aspects concerning professional groups that are not permanently integrated into a specific ward.
2.	Directory knowledge	General knowledge about cause-and-effect relationships. Refers to processes and describes the ‘how’ of things and events. It is operational knowledge, knowledge about causal-analytical relationships.
2.1.	Task accomplishment	Referring to the process by which members of the organisation perform their duties (patient care).
2.1.1.	Team coordination	Refers to the coordination and implementation of patient care on the ward by both ward-based and ward-independent professional groups.
2.1.1.1.	Interfaces	Refers to synergies, boundaries, and overlapping responsibilities between professional groups in the organisation of work and delivery of patient care. The category includes interfaces involving at least two different professional groups.
2.1.1.2.	Patient-centredness	Refers to the practical alignment of work priorities with patients’ well-being.
2.1.1.3.	Responsibilities	Refers to areas of work that require clearly defined responsibilities.
2.1.1.4.	Technology-mediated communication	Refers to communication channels that use technological tools to support coordination and information exchange between professional groups.
2.1.1.5.	Face-to-face communication	Refers to communication channels based on direct, face-to-face interaction for coordination between professional groups.
2.1.1.6.	Time management	Refers to the use and availability of time resources for interprofessional collaboration and patient care.
2.1.1.7.	Admission and discharge	Refers to the coordination of interfaces with external service providers, as well as to tasks related to patient admission and discharge planning within the hospital.
2.1.2.	Quality	Refers to outcomes in patient care that were or were not achieved through interprofessional collaboration.
2.3.	Relationships among people	Referring to the way people relate to and interact with each other within the company. It encompasses both work-related and social interactions—personal encounters that are necessary to perform a task, as well as voluntary or desired interactions with people.
2.3.1.	Responsibility	Refers to the expectation that individuals take responsibility for fostering effective collaboration, both in their own actions and in their expectations of others.
2.3.2.	Familiarity	Refers to the degree of personal familiarity between individuals within the work context.
2.3.3.	Team cohesion	Refers to the development of a shared sense of group belonging, accompanied by mutual assistance and support across professional groups.
2.3.4.	Coping strategies	Refers to how conflicts, problems, and errors are addressed within the hospital.
2.3.5.	Hierarchies	Refers to hierarchical relationships between professional groups within the same organisational level.
2.3.6.	Interaction and communication norms	Refers to expectations regarding how individuals should interact and communicate with one another.
2.3.7.	Interactions with relatives	Refers to how relatives are perceived and how staff interact and communicate with them.

Data collection and full coding were conducted by the first author, who has a background in public health and was a doctoral student at the time of the study. The broader research team included supervisors and researchers with backgrounds in health management, psychology, nursing and medicine. Reflexivity was supported through interprofessional research workshops in which emerging categories, coding decisions and preliminary interpretations were discussed with researchers and professionals from social work, sociology, philosophy, psychology, public health, medicine and linguistics backgrounds.

Inter-coder reliability of three interviews was conducted with the third author, who has a medical background and carried out to verify reliability in a stepwise manner^1^. Agreement increased across all three analytic steps. For Interview 1, agreement rose from 19.23% at the level of raw agreement to 42.42% after technical adjustments and to 92.31% after consensus-based resolution of content-related discrepancies. For Interview 2, the corresponding values were 18.63%, 58.99%, and 98.17%, and for Interview 3, 18.43%, 58.26%, and 96.10%, respectively.

## 3 Results

All 15 planned group discussions were successfully conducted across the five participating facilities, with three discussions per facility at hospital management, ward management, and staff level. In total, 76 individuals participated (47 women, 29 men), thereby reaching the planned minimum recruitment target of 75 participants. All discussions were conducted in German, which was the native or working language of the participants. Transcription and qualitative data analysis were also carried out in German to preserve the original wording, contextual meanings and professional terminology used by the participants.

For publication, relevant excerpts and quotations were translated from German into English after completion of the analysis. The translations were prepared with a focus on semantic equivalence rather than literal word-for-word translation. Attention was paid to preserving the meaning of profession-specific terms, organisational descriptions and references to interprofessional work organisation. The translated quotations were reviewed against the original German transcripts by all authors. Where necessary, wording was adjusted to ensure that the English translation accurately reflected the intended meaning of the original statement. Thus, translation was limited to the presentation of findings and did not affect the coding or analytical process.

Across all facilities, participants were distributed across the three organisational levels as follows: hospital management (*n* = 20), ward management (*n* = 29), and staff (*n* = 27) (Table 4). The total duration of the data material was 14 h and 27 min, comprising 4 h and 34 min at hospital management level, 4 h and 47 min at ward management level, and 6 h and 6 min at staff level (Table 5).

**Table 4:** Distribution of study participants by level and profession.

Level	MD	M	N	T	NP	SW	Total
Hospital Management	5	6	4	5	0	0	20
Ward management	0	6	7	8	5	3	29
Staff	0	6	6	7	3	5	27
Total	5	18	17	20	8	8	76

MD, managing director; M, medical; N, nurse; T, therapist; NP, (neuro-)psychologist; SW, social worker.

**Table 5:** Number of participants and duration per each group discussion.

Level	Interview	Number of participants	Duration
Hospital management	K_M	4	73 min
Hospital management	H_W	4	45 min
Hospital management	R_E	4	47 min
Hospital management	S_C	3	58 min
Hospital management	O_M	5	51 min
Ward management	K_A	5	45 min
Ward management	H_P	7	86 min
Ward management	R_H	5	46 min
Ward management	S_K	6	59 min
Ward management	O_K	6	51 min
Staff	K_P	5	38 min
Staff	H_M	6	98 min
Staff	R_P	5	55 min
Staff	S_M	6	61 min
Staff	O_S	5	54 min
Total		76	867 min

More than 75% of participants were between 30 and 59 years old, and 74% worked between 30 and 40 h per week. Work experience within the respective facility varied: 28 participants had worked there for up to 6 years, 21 for between 6 and 18 years, and 27 for more than 18 years (Table 6).

**Table 6:** Sociodemographic data.

Variable	*n*	%
Citizenship		
German	72	94.73%
Others	4	5.26%
Gender		
Female	47	61.82%
Male	29	38.15%
Divers	0	0%
Age		
18–20 years	0	0%
21–29 years	11	14.47%
30–39 years	17	22.36%
40–49 years	17	22.36%
50–59 years	24	31.57%
60 years or older	7	9.21%
Professional position		
Employee	27	35.53%
Ward manager	29	36.16%
Hospital manager	20	26.32%
Professions in the clinic		
Nurse	17	22.36%
Therapist	28	36.84%
Physiotherapist	8	10.52%
Occupational therapist	8	10.52%
(Neuro-)psychologist	8	10.52%
Speech therapist	3	3.94%
Sports scientist	1	1.31%
Medical	18	23.68%
Social worker/Discharge management	8	10.52%
Managing director	5	6.57%
Work experience in the clinic		
Less than 3 years	12	15.78%
3–6 years	16	21.05%
6–9 years	5	6.57%
9–12 years	6	7.89%
12–15 years	9	11.84%
15–18 years	1	1.31%
18–21 years	8	10.52%
More than 21 years	19	25.00%
Weekly working hours		
Up to 10 h	0	0%
10–20 h	0	0%
20–30 h	2	2.63%
30–40 h	56	73.68%
Over 40 h	18	23.68%

Selected transcript excerpts are included to illustrate the main findings. Quotations are referenced using the following scheme:

“Speaker abbreviation: [shortened quotation]. Clean verbatim quotation” (facility_group discussion, paragraph number in the transcript, organisational level [hospital management, ward management, staff], speaker’s professional group).

### 3.1 Dictionary knowledge—ward structure

The facilities examined had different ward structures. In addition to single-phase wards exclusively for ENR patients in phase B, there were mixed wards that cared for patients in phases B, and C or B–D. A key challenge was that the health status of ENR patients often changed throughout the day. This meant that care requirements were often difficult to assess at the start of the day:

“O13: […] in early rehabilitation. And that’s not as easy to plan as with patients who are fitter and can keep their appointments. And then you take a look. And if they're not awake, then I have nothing to work with. And that’s why I always think it’s good when all the other therapists or the nursing staff or the doctors are there to ask, yes, what happened today? How were the patients? And it fluctuates a lot in early rehabilitation, yes.” (O_S, 50, staff, O13-(Neuro-)Psychology).

**Figure 1:**
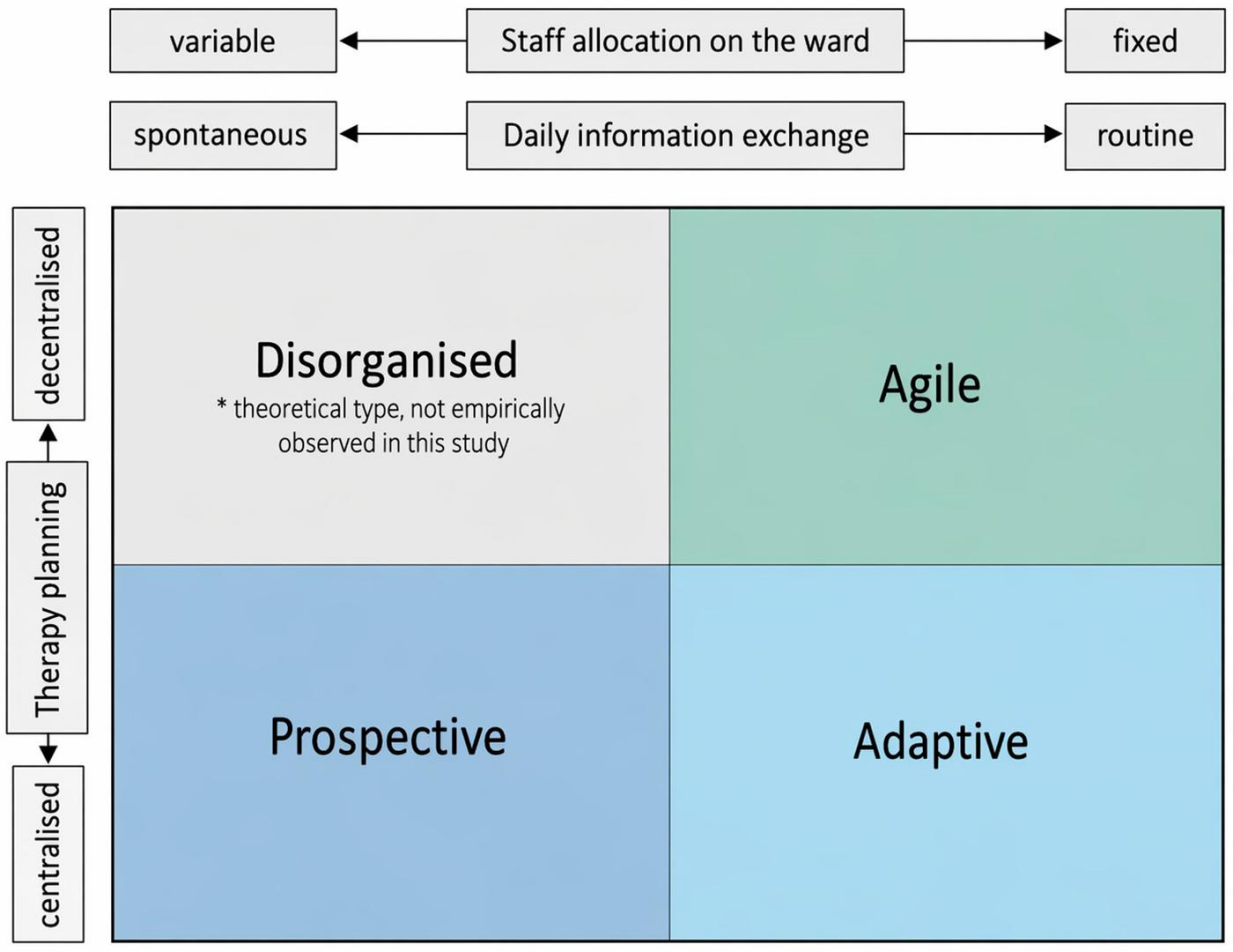
Patterns of work organisation.

This account highlights that interprofessional work organisation in ENR depended not only on patients' fluctuating health status, but also on the underlying organisational structures. Three structural aspects were particularly important: (1) therapy planning, (2) staff allocation, and (3) information exchange on the health status of ENR patients (Figure 1).

#### 3.1.1 Therapy planning

Therapy planning was carried out either centrally by a clinic coordination team or, at the ward level, by an interprofessional ward management team.

Central planning was characterised by fixed therapy time slots and a cross-clinic allocation of therapists. Any adjustments had to be communicated to the central office. Study participants described this as limiting their ability to react at short notice and leading to differences in the level of information available between professional groups.

“K7: […]. We then post the therapy plan, which is posted the day before at around 4 p.m., and our therapists then adhere to this therapy plan. To be honest, we look at where, when, how and who is there from the therapists. But we can’t rely entirely on the plan because very often it is rescheduled and replanned afterwards. […].” (K_A, 27, Ward management, K7 Nursing).

Decentralised planning was organised by the therapeutic ward managers and included daily coordination between therapy and nursing. This enabled short-term adjustments to the health status of ENR patients but required more intensive coordination.

“H2: But the daily appointments, this timetable, what everyone has, that’s clearly a therapeutic ward manager matter.” (H_M, 538, Staff, H2 Therapy).

#### 3.1.2 Staff allocation on the ward

In nursing and medicine, there were predominantly fixed ward assignments. In the therapeutic professions, there were two models. On the one hand, a fixed ward assignment, which favoured stable interprofessional teams, and on the other hand, a variable, cross-ward assignment, which led to frequently changing responsibilities:

“O3: On ward [name 3], all therapy units are planned via therapy planning. This means that we basically have a new therapy plan at least once a day, if not more than once a day. Whereas on ward [name 1], block therapies^2^ are planned, at least as far as occupational and physiotherapy are concerned, which then have fixed times on the ward, usually with a fixed team. And physical therapy, speech therapy and neuropsychology are added on with different time slots, but that is at least a smaller group of people. That means there isn't as much confusion as on ward [name 3], where it is planned across the whole hospital.” (O_K, 111, Ward management, O3 Medicine).

#### 3.1.3 Information exchange

Information was shared both in writing and verbally. Written information was shared via electronic documentation systems, e-mails, digital and analogue patient files, and analogue notices. According to the study participants, difficulties arose regarding the completeness, accuracy, and timely use of existing documentation.

“K4: […]. But in my opinion, we also use e-mail, telephone, [administration programme] a lot to set up all these communication channels again. So it’s not just everything in direct conversations, but also sometimes via e-mail as information or, hopefully soon, even more via [administration programme], so everything is already in there, but sometimes it is not yet used sufficiently by the employees. […]” (K_M, 22, hospital management, K4 Managing Director).

Verbal communication took place spontaneously, for example, through brief discussions or telephone enquiries, as well as within the framework of routine exchange formats. Spontaneous communication was described as fundamentally uncomplicated but was not taken sufficiently into account for the interprofessional work organisation in the care of ENR patients.

“R11: […] our meetings sometimes just take place between doors.” (R_P, 42, Staff, R11 Therapy).

The routine formats included weekly team meetings (ICF meetings) to fulfil the structural requirements of documentation and goal definition. The study participants reported that participation in ward-independent professional groups (e.g., social services, psychology) was difficult in some cases. In several institutions, additional informed deputies were appointed, or preparatory professional group-specific forms were used to reduce information asymmetries. The mandatory weekly team meeting was considered insufficient for daily interprofessional ward work. Therefore, in some institutions, a daily morning interprofessional handover between nursing, therapy, and medicine, and ward-based briefings between nursing and therapy were introduced for interprofessional planning of interface tasks:

“H6: […]. So the day begins with ward meetings, which are attended by both nursing representatives and therapy representatives, and everything is discussed for each patient: Who? What? How was the night from a nursing perspective? The therapists say what the patients can and cannot do, what goals could be achieved and what could not. This is just a rough outline, because for specific goals we also have ICF meetings once a week, where we go through everything again. […]” (H_M, 16, Staff, H6 Medicine).

The combination of therapy planning, staff allocation, and information exchange resulted in distinct patterns of interprofessional work organisation on the wards. The following section examines these patterns from the perspective of directory knowledge, focusing on task accomplishment and relationships among staff.

### 3.2 Directory knowledge—task accomplishment and relationship among people

To manage the daily demands of ward work under the conditions described above, three distinct patterns of interprofessional work organisation emerged from the data: agile, adaptive and prospective work organisation. A fourth theoretical type (disorganised) was used for differentiation purposes but was not observed empirically.

#### 3.2.1 Agile work organisation

The agile approach was characterised by decentralised therapy planning, fixed ward assignments for all professional groups, and routine weekly and daily coordination meetings. A key feature was a structured joint briefing between nursing and therapy staff, during which tasks, responsibilities, and nursing-therapy plans were adapted daily to the current health status of the ENR patients.

“H17: So basically what used to be called a briefing, or sometimes a briefing in the early rehabilitation wards, is about nursing, therapy, where can we help each other? How far along is the patient?” (H_P, 122, Ward management, H17-Medicine).

Study participants described a fast, direct, and spontaneous exchange of information, a high level of mutual support through the possibility of iteratively adjusting treatment plans and tasks, and regular clarification processes when dealing with interface issues. To this end, the wards use an analogue planning board, which was discussed jointly. According to the study participants, this promoted shared responsibility and a high level of mutual support:

“H15: And that’s just, it’s more personally patient-oriented and that’s/that’s what we work for, and that’s why we're here, to be there for the patients, and when you talk about it with each other, then it works much better to get through the day.” (H_P, 197, Ward management, H15 Therapy).

Challenges related to coordinating common time slots and different prioritisation of the IPC between the professional groups.

#### 3.2.2 Adaptive work organisation

The adaptive form was based on centralised therapy planning combined with a fixed ward assignment of the therapy professions. Information was routinely shared weekly, both to specific professional groups and as part of joint interprofessional morning handover meetings. The study participants described a clear distribution of tasks, defined responsibilities, and complementary cooperation between the professional groups. In addition to the daily synchronisation of information for task completion, the study participants also felt that the daily exchange provided psychosocial relief:

“K10: But I think in the mornings, this team also gives you space to say, ‘Wow, this patient is really exhausting’ or ‘The relatives are a bit exhausting at the moment, more complicated or something’. So I also have the impression that you can briefly unburden yourself in the team, because we all know the patients, we also know the relatives who are involved, so you can maybe unburden yourself emotionally a little bit, exactly.” (K_P, 66, Staff, K10-(Neuro-)Psychology).

At the same time, it was reported that the need for coordination in the event of short-term changes in the health status of ENR patients throughout the day remained high and was unstructured. According to the study participants, this resulted in misunderstandings about responsibilities and interprofessional tensions:

“K11: […]. Well, I think that’s most likely a conflict because agreements simply don’t work when there are three parties involved. (laughs)

K13: And as I said, a lot of this has to do with planning and whether it is communicated, […] if it’s not//transferred, if it’s passed on, if it’s passed on to the right place, there’s always a lot, a lot of potential for learning.” (K_P, 102-108, Staff, K11 Therapy, K13 Nursing).

#### 3.2.3 Prospective work organisation

The prospective form was characterised by centrally structured advanced planning of therapy with variable ward assignments for the therapeutic professions. The health status of ENR patients was routinely communicated weekly in team meetings, while daily information was communicated exclusively on an *ad hoc* basis and specific to each professional group. According to study participants, regular rescheduling of therapy led to frequent changes in therapeutic responsibilities, which made it difficult to provide continuity of care for ENR patients. The reliance on individual coordination in everyday ward life led to information asymmetries, repeated information gathering and communication barriers, as well as uncertainties in the interaction between professional groups. Study participants describe this as follows:

“O3: … quite a bit more difficult/Exactly, because every therapist, and there are a lot of therapists compared to block therapies, has to check which nurse is responsible on that day. They have to check where she is. They may or may not get the information/

O1: (talking at the same time) They have to find out about the patient themselves. (They don’t know them either) (talking at the same time)

O3: And vice versa, of course, I have questions like ‘How far have we got with the swallowing assessment?’ Then, of course, if I don’t see an occupational therapist in the corridor, I ask the nursing staff in charge. The nursing staff say, ‘Who was there today?’ And then they think about it until they get feedback about the occupational therapy.” (O_K, 113-115, Ward management, O3 Medicine, O1 Nursing).

#### 3.2.4 Disorganised work organisation

A disorganised type, characterised by decentralised planning, a lack of fixed staff assignments, and exclusively spontaneous information sharing, was not empirically observed in the data material, but was used as a theoretical reference type to distinguish the three empirical forms more clearly from one another.

## 4 Discussion

Across all facilities, three key structural characteristics of interprofessional work organisation were evident on wards caring for ENR patients: the form of therapy planning, staff allocation on the ward, and the organisation of information exchange. Their interplay shaped everyday coordination processes and influenced how IPC was conducted in practice. Where daily coordination routines were well established, study participants described greater confidence and smoother interprofessional interactions. Where responsibilities changed, information was fragmented, or processes remained unclear, they reported uncertainty, reluctance, and a greater potential for conflict.

These findings indicate that ward-level structures are central to the organisation of interprofessional work in ENR. Therapy planning, staff allocation, and information flow not only influenced work processes, but also shaped how professionals related to one another in everyday ward life. In this regard, the findings are consistent with research describing organisational structures as important determinants of effective IPC in complex care contexts. Other authors have shown that IPC is particularly successful where standardised structures and processes are supported by sufficient human, spatial, and temporal resources, as well as by clear roles, fixed teams, and structured communication formats (6, 9–13, 15). More specifically, the present study showed that centralised therapy planning closely tied interprofessional work organisation to fixed time slots. In everyday care, this often led to tensions, particularly when short-term changes in the health status of ENR patients had to be communicated back into central planning structures. By contrast, decentralised ward-based therapy planning appeared to facilitate more flexible adaptation to patients' daily needs. This finding supports previous work from Franz et al. (6) in context of ENR emphasising the relevance of organisational conditions for responsive interprofessional care. At the same time, it further specifies this relationship for ENR by showing how fixed planning structures can become a source of tension when patient conditions fluctuate substantially throughout the day.

Regarding staff allocation, the results showed that nursing and medical professionals were usually embedded in stable ward teams, whereas therapeutic professions were less consistently assigned to specific wards under centralised planning arrangements. For ward-independent professions such as social services or (neuro)psychology, regular participation in team communication and access to up-to-date information proved particularly difficult. A fixed staffing structure on the ward therefore appeared to support interprofessional work organisation. These findings align with other authors highlighting the importance of role clarity, fixed team structures, and stable working relationships for IPC (10–12). They are also supported by Wener et al. (28), who showed that interprofessional collaboration does not emerge automatically from the mere integration of different professional groups, but develops through relationship-building, role clarification and growing reciprocity over time. At the same time, Wener et al. (28) underline that physical proximity alone is not sufficient to ensure functioning IPC, as also emphasised by Wei et al. (18). Rather, continuity of professional presence and reliable integration into communication processes appear to be crucial.

The importance of information exchange was particularly evident in this study. The health status of ENR patients changed frequently, creating a continuous need for interprofessional synchronisation. Although the use of multiple communication channels potentially improved the information base, it also increased coordination effort. Spontaneous consultations alone were vulnerable to misunderstandings, workflow interruptions, and interpersonal tension. By contrast, routine formats such as daily handovers and interprofessional briefings provided reliable structures for planning, decision-making, and mutual support, including psychosocial support. These findings are consistent with previous studies that describe interprofessional communication as a core requirement of collaborative care and at the same time as a potential source of stress (6, 10, 29). The analogue planning board used in one facility also fulfilled functions similar to the electronic boards described by Prætorius et al. (14) as central coordination tools for interprofessional teams. In addition, our findings resonate with Stühlinger et al. (30) who showed that a shared language among healthcare workers is associated with better interprofessional collaboration, higher quality of care, and greater job satisfaction. In this context, psychological safety appears to play an important mediating role, as it may support open communication, shared understanding, and constructive exchange within interprofessional teams.

A similar pattern emerged regarding weekly and daily coordination formats. Although the weekly IPC team meeting fulfilled formal requirements, study participants did not consider it sufficient for implementing care goals in everyday ward practice. Instead, daily interprofessional coordination formats appeared to be necessary to translate overarching goals into ongoing action. This observation reflects Wade's (8) argument that goal setting remains insufficient without continuous coordination of processes and relationships. This interpretation is supported by Lampart et al. (31), who described goal setting as a process that helps coordinate and structure rehabilitation and emphasised the importance of open communication and interprofessional collaboration for overcoming barriers in this process. Their findings also highlight the relevance of institutional structures and culture for collaborative planning, which corresponds with our observation that regular planning and review formats were particularly important in ENR, where patient trajectories were dynamic and goals often had to be revised at short notice. It is also in line with the findings of Franz et al. (6), Goldman et al. (10) and Reeves et al. (32), all of whom emphasise the practical relevance of routine communication formats in supporting collaborative work. The present study adds to this literature by highlighting the dual function of daily meetings: they served not only to coordinate tasks, but also to provide psychosocial support within the team. This is also consistent with Van der Veen et al. (33), who showed that team meetings in neurorehabilitation require sufficient psychological safety to enable constructive conflict, shared decision-making and team learning.

The empirically identified forms of work organisation described as agile, adaptive and prospective can be understood as different responses to these structural conditions. More broadly, the findings suggest that the form of interprofessional collaboration should be aligned with the severity of patients' conditions and that ward-level work organisation should be structured accordingly. In this sense, the three organisational forms offer a useful heuristic model for structuring wards and organising work in both hospitals and rehabilitation facilities. Agile work organisation was characterised by decentralised planning, fixed interprofessional teams, and daily structured coordination. The findings suggest that this form was particularly functional in contexts where patient care required continuous adjustments throughout the day, especially on wards with a high proportion of phase B patients. Adaptive work organisation combined centralised therapy planning with fixed ward assignments for staff. This arrangement appeared to provide clearer role allocation and supported adjustments to therapy procedures, provided that changes in patients' condition were routinely communicated. However, it was also associated with a high coordination effort when care plans had to be modified at short notice. Prospective work organisation, in contrast, was characterised by highly centralised forward planning, variable therapist allocation, spontaneous handovers, and limited shared time slots. Under these conditions, teams faced substantial challenges in responding flexibly to short-term care needs.

The previously described forms of work organisation show clear parallels to Prætorius et al. (14), who argue that standardised structures may be sufficient in predictable contexts but need to be supplemented by flexible interprofessional coordination in highly dynamic situations. The present findings support this view and further suggest that agile work organisation is particularly suited to ENR contexts with high care complexity and daily instability, whereas adaptive and prospective forms are more compatible with predictable care requirements in later rehabilitation phases. This interpretation is further supported by Fagerdal et al. (34), who identified roles, procedures, and the organisation of work, alongside competence and team culture, as central team factors enabling adaptive capacity in hospital teams. From this perspective, the differences identified in therapy planning, staff allocation, and information exchange can be understood not merely as structural variations, but as factors shaping how flexibly teams were able to respond to changing patient needs in everyday practice. In this sense, the findings indicate that organisational structures should be aligned with the severity and variability of patients' conditions. The more unstable and care-intensive the situation, the closer and more coordinated IPC needs to be. This interpretation is also consistent with Franz et al. (6) findings on interprofessional communication in ENR.

Finally, the findings show that organisational structures affected not only task accomplishment but also interpersonal relationships. Where roles, responsibilities, and coordination processes were clear, study participants described trustful collaboration and constructive conflict management. Where structures were unstable or ambiguous, they reported uncertainty, barriers, increased stress, and conflict. This is consistent with previous research identifying organisational ambiguity and role conflict as risk factors for IPC (35–37). These findings also align with recent evidence from neurorehabilitation showing that strong team identification alone does not necessarily ensure effective interprofessional collaboration when psychological safety is limited. Van der Veen et al. (33) found that reduced psychological safety can hinder constructive conflict, shared decision-making and team learning. In this regard, the present findings suggest that stable ward-based team composition, clear responsibilities and routine daily interprofessional briefings may provide reliable spaces in which uncertainties, divergent assessments and coordination needs can be addressed more openly. At the same time, the findings support Wade's (8) assumption that changes to organisational structures may also improve interpersonal relationships. In this sense, IPC in ENR should not be understood solely as a matter of individual attitudes or communication skills, but also as a structural achievement of ward organisation.

### 4.1 Strengths and limitations

A major strength of this study is its interprofessional perspective on structural elements of IPC in ENR. Including different professional groups across several clinics enabled a differentiated analysis of organisational mechanisms and interprofessional negotiation processes. The qualitative design provided an in-depth understanding of these dynamics; however, it does not allow for quantitative conclusions regarding the effects of individual structural features.

Several limitations should be considered. Recruitment was supported by key persons at the study sites, and selection bias as well as social desirability therefore cannot be ruled out. In the interviews, participants generally expressed open and positive views on IPC. Critical perspectives were raised less frequently, possibly because individuals with more critical views did not feel addressed by the study or were not personally invited to participate. The discussion atmosphere was assessed differently across group discussions, ranging from open and constructive to reserved and uncertain. After each recording, a feedback round was conducted. These observations were included in the analysis of interpersonal relationships.

Although the questionnaire was pre-tested, it cannot be assumed that data collection was exhaustive. As the findings are based on group discussions, they rely on the study participants’ self-descriptions. Moreover, the group discussion method prioritised the participants' systems of relevance, so potentially important topics may not have been addressed due to recall bias or social desirability. To support the reliability of the findings, intercoder agreement was successfully established.

In addition, the COVID-19 pandemic constituted a particular contextual condition during the study period and may have influenced practices within the participating facilities. Staff shortages made it difficult to release employees from their regular duties. In addition, site-specific hygiene regulations had to be observed. The group discussions were therefore coordinated individually with each clinic and took place during regular working hours. Consequently, fully anonymous data collection was not possible, as participants were known to each other within their organisational context. To address this, all participants signed a confidentiality agreement before taking part in the group discussions.

### 4.2 Implications

The results show that strengthening IPC in rehabilitation involves not only individual skills or team training, but also structural elements at ward level. These have turned out to be key tools for managing interprofessional work organisation. Team and organizational development are important to strengthen interprofessional collaboration in the future (38).

The data shows that the following structural elements are especially important:

- Stable ward-based assignment of professional staff, particularly therapists, promotes continuity in interprofessional team composition, supports shared routines, and enables ongoing assessment of phase B patients' changing clinical condition.
- Adapting therapy planning to the clinical volatility of ENR patients supports the interprofessional adaptability of work organisation in the face of frequent daily changes in the health status of ENR patients. This requires decentralised or at least flexibly adaptable central therapy and care planning.
- In addition to weekly team meetings, routine daily interprofessional coordination formats (e.g., briefings, handovers) facilitate joint work organisation and reduce information asymmetries. This can help to avoid misunderstandings in everyday work, especially at interfaces between the nursing and therapy professions.

The results also show that structural adjustments in ENR not only have organisational implications but also shape interpersonal relationships. Future research should examine the long-term effects of targeted structural interventions on interprofessional collaboration. These interventions may include the introduction of agile models of work organisation, structured briefings, interprofessional planning boards, the reconfiguration of team meeting structures, and alternative approaches to staff allocation. Further studies should also assess the extent to which such interventions influence the quality of care, treatment outcomes, and patient satisfaction. A systematic comparison between ENR and later rehabilitation phases (C and D) would also help to determine the need for patient-oriented ward design in a more differentiated manner.

## Data Availability

The original contributions presented in the study are included in the article/Supplementary Material, further inquiries can be directed to the corresponding author.

## Footnotes

^1^Stepwise manner of Inter-coder reliability: first, by determining raw agreement; second, by applying technical coding adjustments, particularly regarding segment; and third, by resolving content-related discrepancies through consensus. In cases of multiple possible category assignments, the thematically predominant issue was used as the decision criterion.

^2^The term ‘block therapy’ was introduced by the institution itself and describes a specific form of ward organisation: treatment units are scheduled within a fixed time window for a defined group of patients, without a predetermined sequence or timing.
