# Supplementary material for: Structural conditions of interprofessional collaboration in neurological early rehabilitation wards: a qualitative study
Source: Front Health Serv. 2026 Aug 4;6:1862640. doi: 10.3389/frhs.2026.1862640 (PMC13481838; doi:10.3389/frhs.2026.1862640)
Supplement: Supplementary file 1 [file Table1.docx]

# Structural conditions of interprofessional collaboration in neurological early rehabilitation wards: a qualitative study.

L. Redzewsky Faure^1^, U. Thielhorn^2^, A. Frangos^3^, C.-W. Wallesch^4^, M. Körner^5^

^1^Medical Psychology and Medical Sociology, Faculty of Medicine, University of Freiburg, Freiburg, Germany

^2^Catholic University of Applied Sciences Freiburg

3Institute of General Pracitice, Medial Faculty and Medical Center, University of Freiburg, Germany

^4^BDH-Bundesverband Rehabilitation

^5^ Departement of Health Professions, Institute for Collaborative Practice and Leadership in Healthcare, Bern University of Applied Sciences, Bern, Switzerland

# Supplements

The following Cading Framework is reproduced from the original work by Liliane Redzewsky Faure. Redzewsky Faure, L. (2026). *Organisation und Kultur der interprofessionellen Zusammenarbeit in der neurologischen Frührehabilitation.* Freiburg: Albert-Ludwigs-Universität Freiburg. doi: [10.6094/UNIFR/279663](https://doi.org/10.6094/UNIFR/279663).

**Coding Framework**

Structure of the coding framework:

1. Level: Deductively derived from Sackmann (1991)
1.1. Level: Deductively derived from Sackmann (1991)
1.1.1. Level: Inductively differentiated from the data
1.1.1.1. Level: Inductively differentiated from the data

To avoid duplicate codes within the coding framework, codes were assigned to the relevant subcategories and transferred from the deductive main and subcategories to the inductively developed subcategories. Due to the small number of coded segments in the categories “recipe knowledge” and “axiomatic knowledge,” no inductive subcategories were developed for these categories.

Quotations: To enhance effective anonymisation, speaker abbreviations and professional titles are omitted from the coding manual.

| Level | Theme | Explanation | Quote |
| --- | --- | --- | --- |
| 1. | Dictionary knowledge | Commonly known knowledge about definitions and descriptions. It refers to the ‘what is’ in a particular cultural environment. |  |
| 1.1. | Organisational goal | Refers to what organisational members perceive as the primary goal of their organisation. It also encompasses the realisation of this goal through concrete activities. It expresses the members’ intention, both in the past and present, to work towards this ideal and achieve the desired outcomes. |  |
| 1.1.1. | Healthcare provision mandate | Refers to the shared goal of the hospital and its staff to provide patients with medical, therapeutic, nursing, and supportive interventions aimed at restoring, improving, or maintaining health and quality of life, as well as to the motivation to align their actions with this objective. | „[…] to ensure optimal care in the best interests of the patient“ (O_K, 28) |
| 1.1.2. | Economic efficiency | Refers to the cost-effective and efficient internal use of resources and services, as well as to statements concerning their economic impact on the hospital. | „[…] to increase the number of occupied beds in order to offset rising personnel costs“ (H_W, 278) |
| 1.1.3. | Market performance | Refers to the organisation’s external image, its successful positioning in the market, and its ability to remain competitive. | „We are a small, relatively small and very close-knit hospital, and therefore offer other advantages that sometimes have to be weighed against each other. However, this does not always work. If someone lives in [nearby city] and can cycle to work, that is, of course, a real advantage .(R_H, 115) |
| 1.1.4. | Collaboration | Refers to the organisational goal of establishing collaboration within the organisation. | „The best outcome—or rather, the long-term goal we have—is that, at some point, they will take their breaks or have coffee together. We discussed last Friday whether this could take place at the nurses’ station, but, to be honest, it will still take a long time. It is already happening on some monitoring wards, although the teams there are smaller” (R_E, 39) |
| 1.2. | Organisational structure | Referring to structural aspects and formal procedures of the organisation. These create the conditions for achieving the main objective and implementing the overall strategy. |  |
| 1.2.1. | Hospital network level | Refers to statements concerning the hospital’s non-profit ownership and the significance of its membership in the hospital network. | “We work for a registered non-profit association and have, so to speak, a common mission statement for all clinics. We have also developed leadership principles, which support us in our communication. We are not a shareholder-oriented company where financial returns are the primary concern. Of course, we still aim to achieve at least a balanced budget, but these principles provide a framework for effective communication.“ (K_M, 35) |
| 1.2.2. | Hospital level | Refers to the structure of collaborative formats within the hospital that extend across wards and professional groups. | “The hospital management meeting is an institutional forum held weekly. It brings together and integrates the hospital’s major professional groups. Topics from different professional fields are discussed, and the meeting therefore represents, to some extent, the multidisciplinary or interprofessional structure of the hospital.“ (H_W, 15) |
| 1.2.3. | Ward level | Refers to the structural organisation of wards within the institution. |  |
| 1.2.3.1. | Ward configuration | Refers to how the ward is staffed and how patients are allocated according to the severity of their condition. | “I think you also have a stable ward-based team. Most staff members usually work on the same ward, which means that information exchange is more up to date.“ (S_K, 299) |
| 1.2.3.2. | Therapy planning | Refers to the formal organisation of therapy on the ward, including how therapeutic interventions are planned, | “And then it starts, just as [Person] already said. You look at which patients can somehow be rescheduled, which appointments can go ahead, which cannot, and which need to be moved to the following day. A great deal of flexibility is required. It becomes particularly difficult when you are working within such a rigid framework and have very little room for manoeuvre on certain days.” (S_M, 399) |
| 1.2.3.3 | Ward meetings | Refers to formally scheduled interprofessional meeting formats on the ward, as well as to the absence of such formats. | “At 9 a.m., the meeting with the physicians takes place, and every Tuesday and Thursday the therapists and physicians meet together.” (H_M, 162) |
| 1.2.3.4. | Team meetings | Refers to the OPS 8-552 structural requirement for a weekly interprofessional team meeting based on the ICF framework. During these meetings, weekly patient goals are defined and further care is discussed. The category includes statements concerning the organisation, implementation, challenges, and perceived benefits of these team meetings. | “I also think that the weekly team meeting is very important because it gives us more time to talk. In everyday ward practice, this often receives too little attention. I therefore consider it essential to have an opportunity for close exchange.” (K_P, 23) |
| 1.2.3.5. | Within professional groups | Refers to meeting formats, descriptions, and specific characteristics within individual professional groups. | “There are meetings within the therapy departments, both within individual professional groups and across different therapy professions.” (O_M, 72) |
| 1.2.3.6. | Ward-independent professional groups | Refers to structural aspects concerning professional groups that are not permanently integrated into a specific ward. | “Those of us in social services sometimes cover several wards, particularly when a colleague is absent. As a result, we experience difficulties accessing the information we need.” (S_K, 72) |
| 1.3 | Organisational strategy | Refers to innovations and approaches used to build and maintain a successful and stable organisation. The category does not concern the organisational goal itself, but rather the means and strategies through which this goal is pursued. |  |
| 1.3.1 | Staff leadership | Refers to managers’ views and approaches regarding how staff should be involved and led, as well as employees’ perceptions of how managerial decisions and measures are implemented. The category also encompasses the formal hierarchical structure across two organisational levels. | “Because we often involve many people at different points rather than simply making decisions, a large number of staff members are brought into the process. […] The advantage is that, once an agreement has been reached, everyone supports the decision.” (O_M, 127) |
| 1.3.2 | Interprofessional collaboration (IPC)  ) | Refers to how interprofessional collaboration (IPC) is understood and strategically implemented within the hospital, with a particular focus on its implementation at ward level. | “[…] I believe this marked the beginning of closer interdisciplinary collaboration. We had already initiated this process earlier, and it also led to restructuring within the therapy services and, among other areas, nursing organisation.” (H_W, 23) |
| 1.4 | Organisational members | Refers to managers’ attitudes towards employees and the nature of their relationships with them. |  |
| 1.4.1. | Staff members | Refers to statements made by managers about staff members. |  |
| 1.4.1.1. | Personnel selection | Refers to the criteria and procedures used to recruit and select staff for employment within the hospital. It also includes statements concerning a lack of applications from qualified professionals. | “Yes, we have had vacant positions for several years, and, quite simply, no applicants at all.” (R_H, 109) |
| 1.4.1.2. | Generations | Refers to views on different generations and their expectations, as well as to approaches to supporting early-career professionals within the hospital. | “[…] Generation Z today has somewhat different expectations regarding work–life balance.” (O_K, 181) |
| 1.4.1.3. | Professional boundaries | Refers to managers’ views on existing professional boundaries between occupational groups within the hospital. | “These are deeply ingrained ways of thinking that develop when people remain primarily within their own profession […]” (H_W, 237) |
| 1.4.1.4. | International healthcare professionals | Refers to views on international staff members working within the hospital. | “[…] who may initially view the hospital merely as an entry point or stepping stone, for example, to obtain a work permit in Germany and complete the required recognition of their professional qualifications before applying for positions elsewhere.” (S_C, 35) |
| 1.4.2. | Patients in Phase B | Refers to statements made by managers and staff members about patients receiving neurological early rehabilitation in Phase B. | “[…] Our patient population is increasingly shifting towards patients with the most severe impairments.” (O_K, 90) |
| 2. | Directory knowledge | General knowledge about cause-and-effect relationships. Refers to processes and describes the ‘how’ of things and events. It is operational knowledge, knowledge about causal-analytical relationships. |  |
| 2.1 | Task accomplishment | Referring to the process by which members of the organisation perform their duties (patient care). |  |
| 2.1.1. | Team coordination | Refers to the coordination and implementation of patient care on the ward by both ward-based and ward-independent professional groups. |  |
| 2.1.1.1. | Interfaces | Refers to synergies, boundaries, and overlapping responsibilities between professional groups in the organisation of work and delivery of patient care. The category includes interfaces involving at least two different professional groups. | “Otherwise, we coordinate with the nursing staff so that we can, for example, transfer the patient together from the bed to the wheelchair, or take over certain tasks when staffing is tight—things like that.” (H_M, 20) |
| 2.1.1.2. | Patient-centredness | Refers to the practical alignment of work priorities with patients’ well-being. | “But, as I said, we are working with people, and it may happen that, for example, a patient develops a fever or another complication, which then requires the entire plan to be adjusted.” (K_A, 29) |
| 2.1.1.3. | Responsibilities | Refers to areas of work that require clearly defined responsibilities. | “And in most cases, you know who is responsible for which patient without having to ask.” (O_K, 45) |
| 2.1.1.4. | Technology-mediated communication | Refers to communication channels that use technological tools to support coordination and information exchange between professional groups. | “But now that the documentation is available in [administrative software], there is less need to explain everything verbally. You can read it and know what the issue is.” (H_M, 307) |
| 2.1.1.5. | Face-to-face communication | Refers to communication channels based on direct, face-to-face interaction for coordination between professional groups. | “Our discussions sometimes simply take place informally in passing.” (R_P, 42) |
| 2.1.1.6. | Time management | Refers to the use and availability of time resources for interprofessional collaboration and patient care. | “Because our therapy sessions are scheduled so tightly. We move from one patient to the next in 30-minute intervals, and sometimes there simply are not five minutes available to wait until someone returns from their break.” (K_P, 100) |
| 2.1.1.7. | Admission and discharge | Refers to the coordination of interfaces with external service providers, as well as to tasks related to patient admission and discharge planning within the hospital. | “Who is now going to give the relatives the information: ‘We need to start discharge planning.’” (R_P, 313) |
| 2.1.2. | Quality | Refers to outcomes in patient care that were or were not achieved through interprofessional collaboration. | “And I mean, since I have been working in early rehabilitation, I have had the impression that we work so well as a team that we are able to move quite a number of patients on to Phase C, for example, which I believe is always very important for the team.” (K_P, 118) |
| 2.2. | Adaptation and change | Refers to how the organisation responds to changes perceived in both its internal and external environment. |  |
| 2.2.1. | Crisis management | Refers to measures taken to restore stability and safety following unforeseen events, as well as to their effects within the hospital. | “Looking back over the past year, and this was also the feedback from these teams, the pandemic brought many negative consequences. However, one positive outcome was that nursing and therapy moved even closer together.” (H_W, 26) |
| 2.2.2. | Resistance | Refers to resistance to decisions made by the organisation. | “[…] It was highly heterogeneous, and it was not possible to predict in advance who would resist and who would not. It was interesting to observe, but some individuals were also extremely difficult to convince.” (R_E, 46) |
| 2.2.3. | Flexibility | Refers to the ability to adapt rapidly to new conditions and situations, as well as to the systematic and iterative capacity to respond to emerging requirements and develop appropriate ways of working through self-organisation. | “So, it is certainly the case that you have to be very flexible here.” (R_H, 55) |
| 2.3. | Relationships among people | Referring to the way people relate to and interact with each other within the company. It encompasses both work-related and social interactions – personal encounters that are necessary to perform a task, as well as voluntary or desired interactions with people. |  |
| 2.3.1. | Responsibility | Refers to the expectation that individuals take responsibility for fostering effective collaboration, both in their own actions and in their expectations of others. | “Either you have a capable physician on the ward who thinks ahead, or a nurse who does so. The first therapist who becomes aware of the situation also needs to think ahead.” (O_M, 81) |
| 2.3.2. | Familiarity | Refers to the degree of personal familiarity between individuals within the work context. | “And I believe that this also strengthens a team when people have the opportunity to get to know one another. You may also learn something about someone’s personal life because you enjoy talking beyond purely work-related matters, and this may make the collaboration function even better.” (K_P, 120) |
| 2.3.3. | Team cohesion | Refers to the development of a shared sense of group belonging, accompanied by mutual assistance and support across professional groups. | “It creates a sense of togetherness: everyone is now on the same page, and then we can get started, so to speak. Everyone does their very best for the patients. I think that reflects a very positive team spirit.” (K_P, 24) |
| 2.3.4. | Coping strategies | Refers to how conflicts, problems, and errors are addressed within the hospital. | “Generally, when something is not going well or causes dissatisfaction, whether involving relatives or interprofessional collaboration, it can be helpful to discuss it openly.” (R_P, 67) |
| 2.3.5. | Hierarchies | Refers to hierarchical relationships between professional groups within the same organisational level. | “What the physicians on Ward [Name 1] do is join the nursing handover in the afternoon, at around 2:30 p.m., simply to listen and pick up on the smaller details of what has happened.” (S_M, 390) |
| 2.3.6. | Interaction and communication norms | Refers to expectations regarding how individuals should interact and communicate with one another. | “You can also call anyone at any time. I therefore think that we have a very open communication culture here.” (K_A, 35) |
| 2.3.7. | Interactions with relatives | Refers to how relatives are perceived and how staff interact and communicate with them. | “How should we respond to certain statements so that the patient or their relatives do not misunderstand what is being communicated?” (O_K, 161) |
| 2.4 | Learning mechanisms | Refers to the process of organisational learning. More specifically, the category addresses two questions: how new knowledge is acquired and how existing knowledge is transferred to staff members who do not yet possess it. |  |
| 2.4.1 | Training and continuing education | Refers to the development, reinforcement, and updating of knowledge and skills for professional qualification and continuing education. | “That is why I am very grateful that we work together and can consider who should provide support and how I can teach my staff these skills, so that we can continue learning together across professional boundaries and make the best possible use of the resources available to benefit our patients.” (R_E, 28) |
| 2.4.2 | Peer to Peer | Refers to learning from colleagues. | “Yes, you simply learn from one another. I have come to realise this over the years, and I would no longer want to work without this interdisciplinary approach because the different professions are so closely interconnected.” (R_P, 31) |
| 2.4.3 | Education and vocational training | Refers to opportunities to complete all or part of a professional training programme within the hospital, as well as to how vocational education prepares trainees for everyday professional practice. | “In terms of how we work, we collaborate well across professions. Above all, we see it as our responsibility to model effective collaboration for the students and to show them how professional groups can work hand in hand, so that they are eventually well prepared to move on to the regular wards and work effectively there.” (H_W, 155–157) |
| 2.4.4 | Job shadowing and induction | Refers to concepts and procedures for integrating new staff members or prospective employees into the organisation. | “As part of the induction process, it is mandatory to spend some time in all areas—not only on the wards, but across all departments—for example, by shadowing physiotherapy staff on the wards, so that new employees become familiar with the different professional areas.” (R_P, 51) |
| 2.4.5 | Reflexion | Refers to the ability and opportunity to critically reflect on past experiences and reconsider established practices. | “Nevertheless, especially because we all have so little time, I think it is important that we repeatedly question our processes […].” (H_M, 609) |
| 3. | Recipe knowledge | Recipe knowledge is defined as cultural knowledge concerning recommendations, improvement strategies, and approaches to addressing problems. It includes understandings of what should be done to improve existing practices and how to respond when something goes wrong. | “In my view, this time slot—or the team meeting itself—could be used more effectively to discuss the patients in greater depth. Sometimes, and this also depends on the staff involved, the discussion is largely limited to questions such as: discharge or no discharge, and where will the patient go? Then the team moves on to the next patient. In my opinion, the substantive discussion is often neglected, even though the time is actually available for it.” (K_A, 25) |
| 4. | Axiomatic knowledge | Refers to knowledge that shapes the foundation and continued existence of organisations, including their identity, aspirations, strategies, structures, and activities. It provides insight into the underlying “why” behind information across different content areas. | “Phase B always comes first and is given the highest priority.” (O_S, 289)  “If the position is a good fit, the hospital is well suited for staying long term. The organisation also makes considerable efforts to retain staff.” (O_K, 185)  “However, many therapists are still strongly attached to thinking in terms of their own departments. I would say that this is also part of the hospital’s legacy […].” (R_E, 45) |
